# Propolis Attenuates Cisplatin-Induced Ovarian Injury by Modulating Oxidative Stress, Inflammation, Apoptosis, and GRP78/ATF6/CHOP Pathway

**DOI:** 10.3390/cimb48020212

**Published:** 2026-02-14

**Authors:** Bakiye Akbaş, Şeyda Kanbolat, Merve Badem, Oktay Yıldız, Mustafa Özgür Yalman, Engin Yenilmez, Rezzan Aliyazıcıoğlu

**Affiliations:** 1 Department of Obstetrics and Gynecology, Faculty of Medicine, Karadeniz Technical University, Trabzon 61080, Turkey; 2 Department of Biochemistry, Faculty of Pharmacy, Karadeniz Technical University, Trabzon 61080, Turkey; seydaakkaya@ktu.edu.tr (Ş.K.); ecz.mervebadem@gmail.com (M.B.); oktayyildiz29@hotmail.com (O.Y.); rezzan@ktu.edu.tr (R.A.); 3 Department of Histology and Embryology, Faculty of Medicine, Karadeniz Technical University, Trabzon 61080, Turkey; md.mustafayalman52@gmail.com (M.Ö.Y.); yenilmez@ktu.edu.tr (E.Y.)

**Keywords:** propolis, cisplatin, ovary, toxicity, cancer

## Abstract

Cisplatin-induced ovarian damage is a significant concern for young women receiving chemotherapy. Although propolis, a polyphenol- and flavonoid-rich natural product, has been proposed as a protective agent, its effects on cisplatin-related ovarian injury remain insufficiently defined. This study aimed to investigate whether propolis mitigates cisplatin-induced ovarian toxicity. In this study, 36 adult female Wistar rats were randomly allocated into six groups: Control, Propolis (50 mg/kg), Propolis (100 mg/kg), Cisplatin (7 mg/kg), Cisplatin + Propolis (50 mg/kg), and Cisplatin + Propolis (100 mg/kg). Cisplatin was administered as a single intraperitoneal dose on day 1, while propolis was given orally by gavage once daily for 14 days. Biochemical, histopathological, and endoplasmic reticulum (ER)-stress-related parameters were evaluated. Histopathologically, cisplatin caused significant vascular congestion, hemorrhage, edema, and follicular degeneration (*p* < 0.01), accompanied by marked reductions in primordial, primary, secondary, and tertiary follicle counts and a significant increase in atretic follicles. Propolis co-administration significantly ameliorated these lesions and partially preserved follicular counts, particularly at the 100 mg/kg dose (*p* < 0.01). Cisplatin markedly increased malondialdehyde (MDA) levels and ER stress markers (GRP78, ATF6, and CHOP), while reducing glutathione (GSH). Propolis treatment ameliorated these changes, decreased TNF-α and caspase-3 levels, and attenuated oxidative, inflammatory, and apoptotic responses. Propolis exerts strong antioxidant, anti-inflammatory, anti-apoptotic, and ER-stress-modulating effects that collectively counteract cisplatin-induced ovarian injury.

## 1. Introduction

Cancer remains a major global cause of death, accounting for 19.3 million new cases and 10 million deaths in 2020 [1]. Its incidence is rising among young adults, especially those of reproductive age. Notably, cancers diagnosed before 50 increased by 79% from 1990 to 2019, with thyroid cancer showing a ~3% annual rise in individuals aged 15–39 years [2,3]. As survival improves, fertility preservation has become a critical priority in younger patients.

Cisplatin is a widely used platinum chemotherapeutic for testicular, ovarian, bladder, and lung cancers [4,5]. Its efficacy is limited by dose-related toxicities—particularly gonadotoxicity—alongside nephrotoxicity, ototoxicity, and neurotoxicity [4]. Clinical and experimental evidence shows that cisplatin disrupts ovarian reserve through oxidative-stress-driven apoptosis and accelerated follicular loss, contributing to premature ovarian failure and infertility [6,7,8,9]. These risks highlight the urgent need for protective strategies during chemotherapy.

Beyond oxidative stress and inflammation, emerging evidence indicates that additional cellular stress pathways may contribute to cisplatin-induced ovarian injury. Although ER stress has been implicated in cisplatin-induced ovarian injury, existing studies have primarily focused on individual components of this pathway [10,11]. A comprehensive evaluation of key ER stress markers, encompassing stress initiation (GRP78), adaptive signaling (ATF6), and ER-stress-related apoptosis (CHOP), in parallel with tissue-level indicators of ovarian injury and follicular integrity, has not been systematically examined within the same experimental framework in ovarian tissue. Importantly, ovarian protection should be interpreted at the tissue level, reflecting preservation of follicular architecture and attenuation of stromal injury, rather than being inferred solely from molecular or biochemical alterations. Addressing these gaps is critical for clarifying the mechanistic basis of cisplatin-induced ovarian toxicity and for identifying effective protective strategies.

Recent studies show that melatonin, resveratrol, and vitamin C–inositol combinations offer only partial protection against cisplatin-induced ovarian damage [12,13,14]. In contrast, propolis—rich in polyphenols and flavonoids—exerts broader antioxidant and anti-inflammatory actions across multiple experimental settings, including ischemia/reperfusion, in vivo ovarian injury, and in vitro ovarian cell models [15,16,17,18,19,20,21].

The biological activity of propolis is attributed to multiple bioactive constituents, notably caffeic acid phenethyl ester (CAPE), chrysin, pinocembrin, and galangin. CAPE is a well-characterized phenolic ester that suppresses Nuclear factor kappa B (NF-κB)-mediated oxidative stress and confers cytoprotection across multiple organ systems [22,23]. Chrysin similarly modulates inflammatory signaling via NF-κB inhibition and has demonstrated protective effects against toxin-induced ovarian oxidative stress, inflammation, and apoptosis [24,25]. Pinocembrin, one of the most abundant flavonoids in propolis, exhibits pronounced neuroprotective properties by limiting neuronal apoptosis, reducing cerebral infarct size, and preserving blood–brain barrier integrity following ischemic injury [26,27]. Likewise, galangin provides multi-organ protection by activating endogenous antioxidant defenses, particularly nuclear factor erythroid 2–related factor 2/heme oxygenase-1 (Nrf2/HO-1) signaling, while suppressing inflammation-associated cell death in cardiac and hepatic injury models [28,29,30]. Collectively, these findings suggest that propolis exerts integrated cytoprotective effects through convergent antioxidant and anti-inflammatory mechanisms across diverse tissues.

Although propolis exhibits protective potential in various experimental models, its role in mitigating cisplatin-induced ovarian injury, particularly through modulation of ER-stress-related pathways, remains unclear. Therefore, the present study investigates the effects of propolis on major pathological processes, including oxidative stress, inflammation, and apoptosis, and specifically evaluates its impact on ER-stress-associated markers GRP78, CHOP, and ATF6 in a cisplatin-induced ovarian injury rat model. By integrating molecular findings with histopathological outcomes, this study aims to clarify the mechanistic basis of ovarian protection and to inform the development of adjuvant strategies for preserving ovarian function during chemotherapy.

## 2. Materials and Methods

### 2.1. Animals

This randomized, controlled experimental study was conducted on 36 adult female Wistar rats (10–12 weeks old, 225–275 g) obtained from the Karadeniz Technical University (KTU) Surgical Research and Application Center, Trabzon, Turkey. Animals were housed under standard laboratory conditions (12 h light/dark cycle, temperature 22 ± 1 °C, humidity 45–65%) with ad libitum access to food and water. All experimental procedures were approved by the KTU Local Ethics Committee for Animal Experiments and performed in accordance with the ARRIVE guidelines and the National Institutes of Health (NIH) Guide for the Care and Use of Laboratory Animals.

### 2.2. Propolis Extract Characterization

The water-soluble propolis extract used in this study was obtained from a commercial source (O’na Propolis, Okta R&D Engineering Services Industry and Trade Co., Ltd., Trabzon, Turkey). The extract had a propolis concentration of 40% and was stored under refrigerated conditions following procurement.

Total phenolic content (TPC) was determined using the Folin–Ciocalteu method as described by Slinkard and Singleton [31]. Briefly, 680 µL of distilled water, 400 µL of 0.2 N Folin–Ciocalteu reagent, 20 µL of the sample, and 400 µL of 10% sodium carbonate were mixed and incubated for 2 h. Absorbance was measured at 760 nm using a spectrophotometer (Evolution™ 201, Thermo Scientific, Waltham, MA, USA). Gallic acid was used as the reference standard, and results were expressed as mg gallic acid equivalents per mL (mg GAE/mL).

Total flavonoid content (TFC) was evaluated according to the method described by Mohammadzadeh et al. [32]. A total of 0.5 mL of sample extract was mixed with 2.15 mL methanol, 0.05 mL of 10% aluminum nitrate, and 0.05 mL of 1 M ammonium acetate. After incubation for 40 min, absorbance was measured at 415 nm. Quercetin was used as the calibration standard, and results were expressed as mg quercetin equivalents per mL (mg QE/mL).

Antioxidant activity was assessed using the ferric reducing antioxidant power (FRAP) assay according to Benzie and Strain [33]. Freshly prepared FRAP reagent (3 mL) was mixed with 0.1 mL of the sample, incubated for 4 min, and absorbance was measured at 593 nm. Results were expressed as mg Trolox equivalents per mL (mg Trolox/mL).

The DPPH (2,2-diphenyl-1-picrylhydrazyl) radical scavenging activity was evaluated using the method described by Molyneux [34]. Equal volumes (0.75 mL) of DPPH solution (0.04 mg/mL) and the sample were mixed and incubated for 50 min. Absorbance was measured at 517 nm, and antioxidant capacity was expressed as SC_50_ values (the concentration required to scavenge 50% of DPPH radicals, mg/mL).

The compositional characteristics of the propolis extract, including TPC, TFC, FRAP, and DPPH–SC_50_ values, are summarized in Table 1.

### 2.3. Experimental Groups and Drug Administration

**Figure 1:**
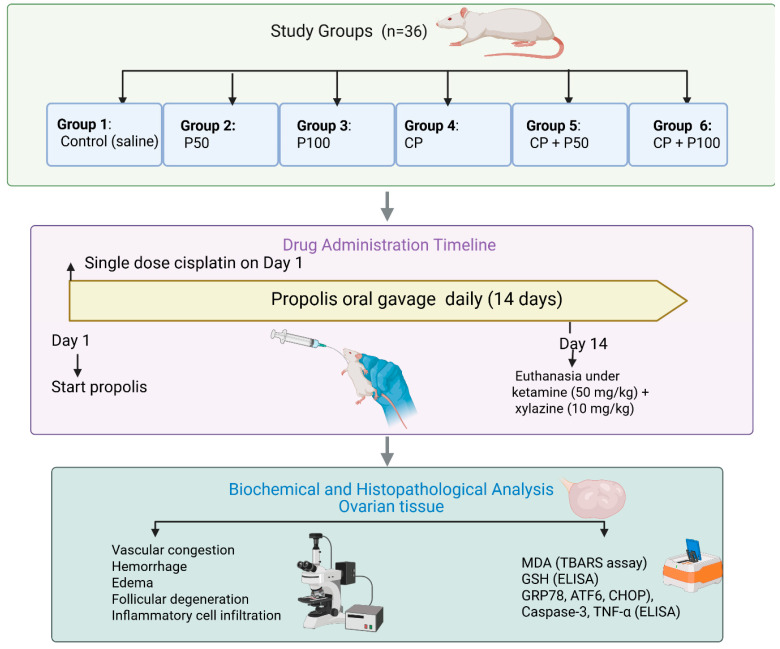
Overview of the study groups, drug administration timeline, and biochemical and histopathological analyses performed on ovarian tissue. A total of 36 female Wistar albino rats were randomly assigned into six groups: Group 1 (Control, saline), Group 2 (P50), Group 3 (P100), Group 4 (Cisplatin), Group 5 (Cisplatin + P50), and Group 6 (Cisplatin + P100).

Animals were randomly allocated into six groups (n = 6 per group) using a computer-generated randomization list (Figure 1):

Group 1 (Control group): Rats received 0.9% physiological saline intraperitoneally (i.p.) once daily for 14 consecutive days.

Group 2 (Propolis 50 mg/kg group): Rats were administered 50 mg/kg propolis orally by gavage once daily for 14 days.

Group 3 (Propolis 100 mg/kg group): Rats received 100 mg/kg propolis orally by gavage once daily for 14 days.

Group 4 (Cisplatin group): Rats were given a single intraperitoneal dose of 7 mg/kg cisplatin on day 1 of the experiment.

Group 5 (Cisplatin + Propolis 50 mg/kg group): Rats received a single i.p. dose of 7 mg/kg cisplatin on day 1, followed by 50 mg/kg propolis administered orally by gavage once daily for 14 days.

Group 6 (Cisplatin + Propolis 100 mg/kg group): Rats were given a single i.p. dose of 7 mg/kg cisplatin on day 1, followed by 100 mg/kg propolis administered orally by gavage once daily for 14 days.

Commercial propolis extract was purchased from BEE’O (Istanbul, Turkey). Cisplatin was freshly prepared in saline before administration.

### 2.4. Anesthesia and Tissue Collection

At the end of the experimental period, animals were anesthetized with ketamine (50 mg/kg, i.p.) and xylazine (10 mg/kg, i.p.). Following bilateral ovariectomy, animals were euthanized by exsanguination via abdominal aorta transection under deep anesthesia. Blood samples were collected, centrifuged at 3000 rpm for 10 min, and stored at −80 °C until biochemical analysis. Ovarian tissues were divided into two parts: one for histopathological and the other for biochemical analyses (Figure 1).

### 2.5. Histopathological Analysis

Ovarian tissues from each group were fixed in 10% neutral-buffered formalin for 48 h and embedded in paraffin after standard tissue processing. Sections of 5 µm thickness were cut using a fully automated rotary microtome (Leica RM2255, Wetzlar, Germany), mounted on slides, and stained with hematoxylin and eosin (H&E) for histological evaluation. Microscopic analysis was performed under 200× and 400× magnification using an Olympus BX51 light microscope (Olympus, Tokyo, Japan), and representative images were captured using an Olympus DP71 camera (Olympus, Tokyo, Japan).

Follicular evaluation was conducted on two sections per ovary, obtained at 50 µm intervals. All fields of each section were screened, and the numbers of primordial follicles, primary follicles, secondary follicles, tertiary follicles, and atretic or degenerated follicles were recorded. The average follicle count from the two sections was used for analysis.

In this study, ovarian follicles were categorized into four developmental stages—primordial, primary, secondary, and tertiary—according to standard histomorphological features reported in the literature [35,36]. Primordial follicles were identified within the superficial cortical region and consisted of a dormant oocyte surrounded by a single layer of flattened granulosa cells. Primary follicles were characterized by a centrally located oocyte enclosed by a single layer of cuboidal granulosa cells. Secondary follicles displayed multiple layers of cuboidal granulosa cells without formation of an antral cavity. Tertiary (antral) follicles were distinguished by the presence of a fluid-filled antrum, a well-organized multilayered granulosa compartment, and clearly developed theca interna and externa layers. Atretic follicles were identified by the presence of oocyte degeneration or loss accompanied by pyknotic and disorganized granulosa cells on hematoxylin–eosin–stained sections.

In addition, five randomly selected high-power fields (200× or 400×) per section were examined to assess histopathological changes. Tissue injury was scored semi-quantitatively for vascular congestion, hemorrhage, follicular degeneration, inflammatory cell infiltration, and interstitial edema based on predefined morphological criteria. Follicular degeneration was defined by the presence of apoptotic cells in the follicular lumen, nuclear pyknosis and cellular atrophy in granulosa cells, oocyte membrane shrinkage, and increased cytoplasmic vacuolization.

For all histopathological parameters, scoring was based on the extent and distribution of the observed lesions as follows: score 0 indicated absence of the lesion; score 1 (mild) represented focal involvement affecting less than 25% of the examined field; score 2 (moderate) indicated multifocal involvement affecting approximately 25–50% of the tissue; and score 3 (severe) corresponded to diffuse involvement affecting more than 50% of the examined area [37,38].

Histopathological evaluations were performed by an experienced histologist blinded to the treatment groups.

### 2.6. Biochemical Analyses

#### 2.6.1. Tissue Homogenization

Approximately 1 g of ovarian tissue from each rat was minced with a sterile scalpel and rinsed in 0.5 mM phosphate buffer (pH 7.4). Samples were blotted dry, placed in numbered tubes, and diluted with nine volumes of the same buffer. Homogenization was carried out at 16,000 rpm for 30 s, with the device rinsed between samples. The homogenates were centrifuged at 1000× *g* for 30 min at 4 °C, and the supernatants were stored for biochemical assays.

#### 2.6.2. Total Protein Determination

Total protein content was measured by the Bradford method [39]. Coomassie Brilliant Blue G-250 reagent was prepared in ethanol and o-phosphoric acid and filtered. For each sample, 2 mL reagent + 20 µL homogenate were mixed, and absorbance was read at 595 nm. Protein concentrations were calculated from a BSA standard curve (0.2–1.0 mg/mL) and expressed as mg/mL.

#### 2.6.3. Oxidative Stress Markers

Lipid peroxidation was evaluated using the TBARS assay [40]. Supernatants (500 µL) were mixed with phosphoric acid and TBA, incubated at 101 °C for 45 min, centrifuged, and read at 532 nm. MDA concentrations were calculated using 1,1,3,3-tetraethoxypropane standards and expressed as nmol/mg protein. Reduced glutathione (GSH) levels were measured using a commercial ELISA kit.

#### 2.6.4. Er-Stress Markers, Inflammatory and Apoptotic Markers

CHOP, GRP78, and ATF6 levels in tissue homogenates were quantified using commercial ELISA kits according to manufacturer instructions. TNF-α and caspase-3 concentrations were determined using commercial ELISA kits, following the supplied protocols.

#### 2.6.5. ELISA Kits

Commercially available ELISA kits were used for the quantitative determination of CHOP, GRP78, ATF6, TNF-α, caspase-3, and GSH levels according to the manufacturers’ instructions. CHOP levels were measured using an ELISA kit (Cat. No. ESD018R, Lot No. KD854250102136; EsdBiotech, Shanghai, China). GRP78 was analyzed using a commercially available ELISA kit (Cat. No. RE2674R, Lot No. CD852650102137; ReedBiotech, Shanghai, China), while ATF6 levels were determined using an ELISA kit supplied by the same manufacturer (Cat. No. RE1300R, Lot No. KD954850102135; ReedBiotech, Shanghai, China). TNF-α concentrations were quantified using a rat-specific ELISA kit (Cat. No. ABT1060Ra, Lot No. KD609750107813; A.B.T., Ankara, Turkey). Caspase-3 levels were measured using an ELISA kit (Cat. No. ABT2850Ra, Lot No. KD903850107833; A.B.T., Ankara, Turkey), and reduced glutathione (GSH) levels were determined using a commercially available ELISA kit (Cat. No. ABT10155Ra, Lot No. KD636550107806; A.B.T., Ankara, Turkey).

### 2.7. Statistical Analysis

ANOVA with Tukey’s test was used for normally distributed, homogeneous data; non-normal or unequal-variance data were analyzed with the Kruskal–Wallis and Bonferroni-adjusted Mann–Whitney U tests, or with Welch’s ANOVA and Games–Howell when variances differed. Histopathological scores were evaluated using non-parametric methods and reported as median [IQR], while biochemical results were expressed as mean ± SEM or median [IQR]. A *p*-value < 0.05 was considered significant.

## 3. Results

### 3.1. Histopathological Findings of Ovarian Tissue

#### 3.1.1. Histopathological Damage Parameters of Ovarian Tissue

##### Vascular Congestion

Median [IQR] values were: Control: 0 [0–0.25], P50: 0 [0–0.25], P100: 0 [0–1], CP: 3 [2.25–3], CP + P50: 1 [1], CP + P100: 0 [0–1].

**Figure 2:**
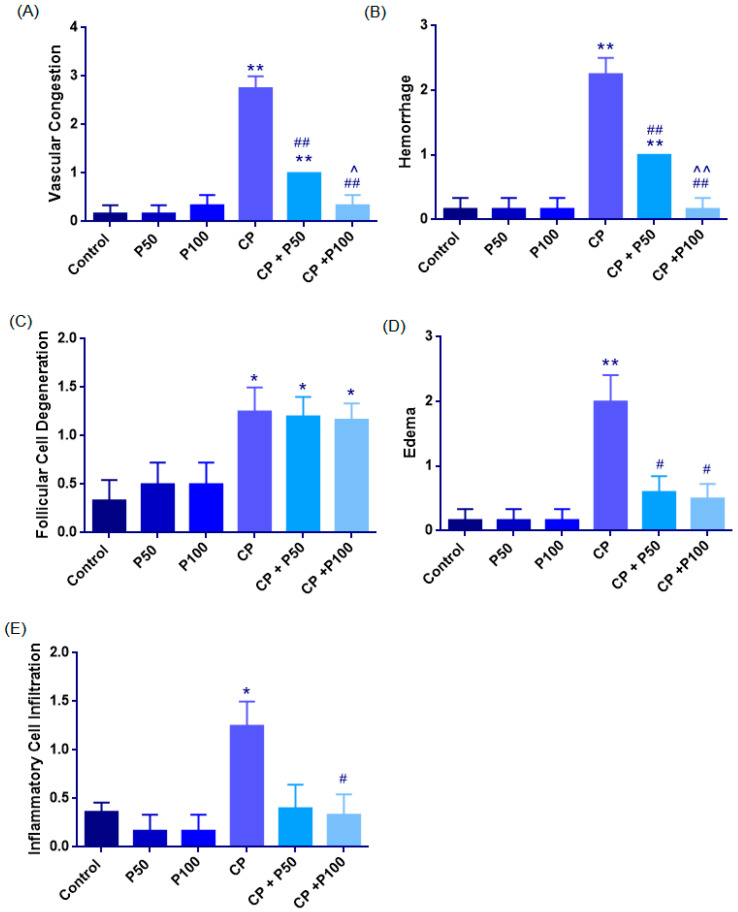
Histopathological damage scores including (**A**) vascular congestion, (**B**) hemorrhage, (**C**) follicular cell degeneration, (**D**) edema, and inflammatory cell infiltration (**E**) across the experimental groups. Data are presented as mean ± SEM. Statistical significance: * *p* < 0.05, ** *p* < 0.01 vs. Control group; # *p* < 0.05, ## *p* < 0.01 vs. CP group; ^ *p* < 0.05, ^^ *p* < 0.01 vs. CP + P50 and CP + P100 groups.

**Figure 3:**
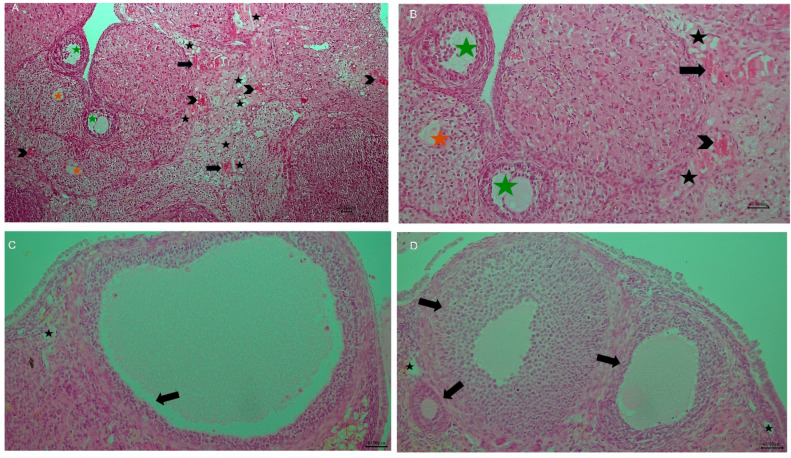
(**A**) Group 4 (Cisplatin, 100×): Vascular congestion (black arrow), hemorrhage (arrowhead), and interstitial edema (black star) are evident. Follicular degeneration (green star) and atretic follicles (orange star) are also present. (**B**) Group 4 (Cisplatin, 200×): Congestion (black arrow), hemorrhage (arrowhead), and edema (black star) persist, accompanied by degenerative follicular changes (green star) and atretic follicles (orange star). (**C**) Group 5 (CP + Propolis 50 mg/kg, 200×): Ovarian follicles (black arrow) show mild degeneration, with mild interstitial edema (black star). (**D**) Group 6 (CP + Propolis 100 mg/kg, 200×): Mild follicular degeneration (black arrow) is observed, and vascular structures (black star) resemble those of the control group. Scale bars: 40 μm in all photos.

**Figure 4:**
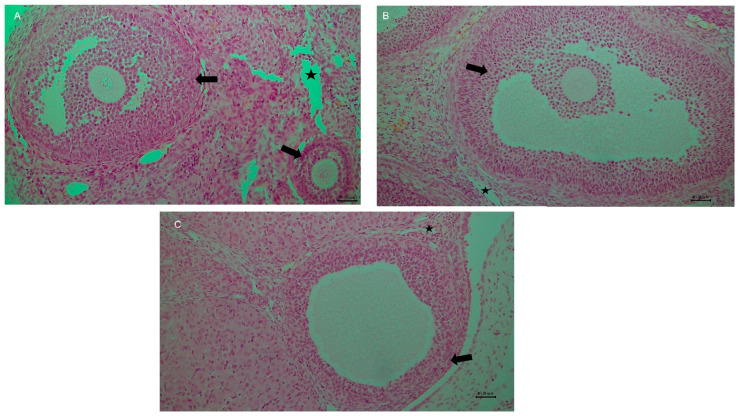
(**A**) Group 1 (Control), 200×: Ovarian follicles (black arrow) show normal morphology, with no vascular congestion (star), hemorrhage, edema, or leukocyte infiltration. (**B**) Group 2 (Propolis 50 mg/kg), 200×: Follicular structures (black arrow) and vessels (black star) appear comparable to the control group, without remarkable pathology. (**C**) Group 3 (Propolis 100 mg/kg), 200×: Follicles (black arrow) and vascular structures (black star) display normal architecture similar to controls. Scale bars: 40 μm in all photos.

**Figure 5:**
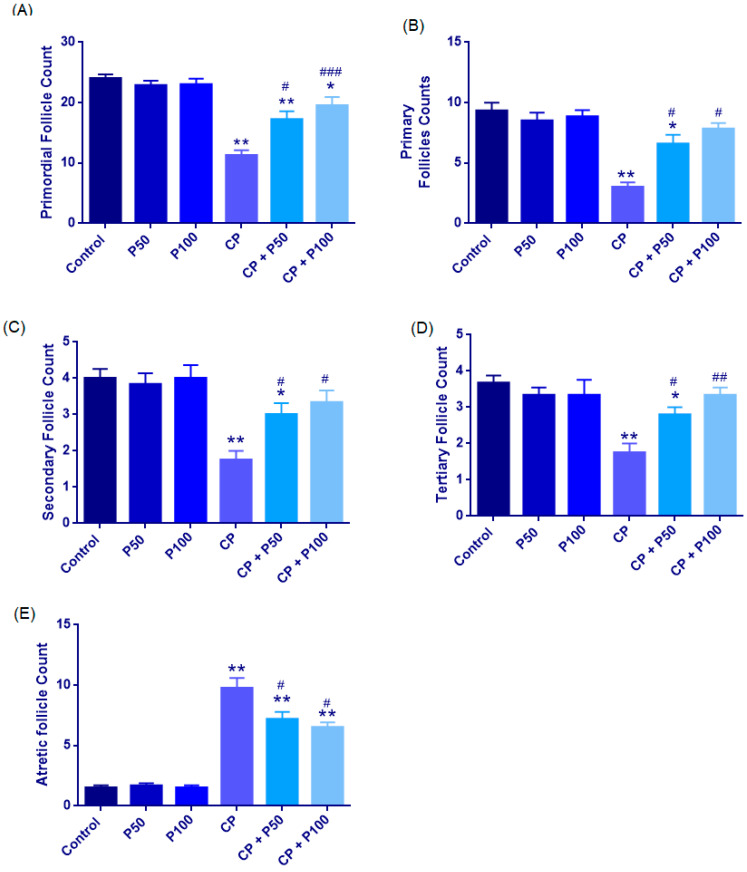
Follicular counts in ovarian tissue including (**A**) primordial follicle count, (**B**) primary follicle count, (**C**) secondary follicle count, (**D**) tertiary follicle count, and (**E**) atretic follicle count across all experimental groups. Data are presented as mean ± SEM. Statistical significance: * *p* < 0.05, ** *p* < 0.01 vs. Control group; # *p* < 0.05, ## *p* < 0.01, ### *p* < 0.001 vs. CP group.

Cisplatin markedly increased congestion compared with the Control group (*p* = 0.006). Propolis co-treatment significantly reduced this effect, with CP + P50 (*p* = 0.006) and CP + P100 (*p* = 0.007) showing lower scores than CP. Control also differed from CP + P50 (*p* = 0.008), whereas no differences were observed between Control and P50, P100, or CP + P100 (*p* > 0.05). CP + P50 and CP + P100 differed slightly (*p* = 0.029). Overall, cisplatin caused severe vascular congestion, while propolis at 50 and 100 mg/kg substantially alleviated this lesion (Figure 2A, Figure 3, Figure 4 and Figure 5; Table 2).

##### Hemorrhage

Median [Iqr] Values Were: Control: 0 [0–0.25], P50: 0 [0–0.25], P100: 0 [0–0.25], CP: 2 [2–2.75], CP + P50: 1 [1], CP + P100: 0 [0–0.25].

Cisplatin significantly increased hemorrhage compared with the Control group (*p* = 0.006). Propolis co-treatment significantly reduced ovarian injury, as both CP + P50 (*p* = 0.006) and CP + P100 (*p* = 0.006) had lower scores than CP, indicating a protective microvascular effect. The Control group differed from CP + P50 group (*p* = 0.008). No differences were observed between Control and P50, P100, or CP + P100 (*p* = 1.000). CP + P100 provided slightly better protection than CP + P50 (*p* = 0.008) (Figure 2B, Figure 3 and Figure 4; Table 2).

##### Follicular Cell Degeneration

Median [IQR] values were Control: 0 [0–1], P50: 0.50 [0–1], P100: 0.50 [0–1], CP: 1 [1–1.75], CP + P50: 1 [1–1.5], and CP + P100: 1 [1–1.25].

Greater follicular cell degeneration was observed in the CP group compared with the Control group (*p* = 0.034). Significant differences were also evident between the Control group and both CP + P50 (*p* = 0.024) and CP + P100 (*p* = 0.018), indicating that propolis co-treatment provided only partial improvement against cisplatin-induced degeneration. Cisplatin–propolis co-treatment did not produce statistically significant reductions when compared with cisplatin alone, although numerical improvements were observed (CP vs. CP + P50: *p* = 0.866; CP vs. CP + P100: *p* = 0.759). Similarly, no significant difference was found between the two propolis co-treatment groups (CP + P50 vs. CP + P100: *p* = 0.892) (Figure 2C, Figure 3 and Figure 4; Table 2).

##### Edema

The Shapiro–Wilk Test showed that edema scores were not normally distributed; Therefore, non-parametric analyses were applied. The Kruskal–Wallis test demonstrated a significant overall difference among the Groups (Χ^2^(5) = 14.842, *p* = 0.011). Median [Iqr] Values Were as Follows: Control: 0 [0–0.25], P50: 0 [0–0.25], P100: 0 [0–0.25], CP: 2 [1.25–2.75], CP + P50: 1 [0–1], and CP + P100: 0.50 [0–1]. CP markedly increased edema compared with control (*p* = 0.008). Propolis co-treatment significantly reduced edema severity, with CP + P50 (*p* = 0.028) and CP + P100 (*p* = 0.019) showing lower scores than CP no difference was detected between the two co-treatment groups (*p* = 0.752).

Control did not differ from P50, P100, CP + P50, or CP + P100 (*p* > 0.05), indicating that propolis alone or in combination did not elevate edema beyond baseline (Figure 2D, Figure 3 and Figure 4; Table 2).

##### Inflammatory Cell Infiltration

Significant differences in inflammatory cell infiltration were observed among the groups (χ^2^(5) = 11.269, *p* = 0.046). Median [IQR] scores were: Control: 0 [0–0.25], P50: 0 [0–0.25], P100: 0 [0–0.25], CP: 1 [1–1.75], CP + P50: 0 [0–1], CP + P100: 0 [0–1].

Cisplatin significantly increased infiltration compared with Control (*p* = 0.013). Propolis co-treatment showed a slight reduction, but this was not significant in the CP + P50 group (*p* = 0.055). CP + P100, however, produced a significant decrease compared with CP (*p* = 0.034). No difference was found between the two co-treatment groups (*p* = 0.827). Control did not differ from P50, P100, CP + P50, or CP + P100 (*p* > 0.05) (Figure 2E, Figure 3 and Figure 4; Table 2).

#### 3.1.2. Follicular Morphology and Quantitative Analysis

##### Primordial Follicle Count

Primordial follicle counts were normally distributed across groups (Shapiro–Wilk, *p* > 0.05), with homogeneity of variances confirmed by Levene’s test (F(5,27) = 1.401, *p* = 0.255). One-way ANOVA showed a significant overall group effect (F(5,27) = 17.486, *p* < 0.001). Tukey HSD analysis revealed that cisplatin markedly reduced primordial follicle counts compared with the Control, P50, and P100 groups (all *p* < 0.001), whereas propolis alone (P50 and P100) had no significant effect versus the Control (both *p* > 0.95).

Co-administration of propolis significantly attenuated cisplatin-induced follicular loss, with higher primordial follicle counts observed in both CP + P50 (*p* = 0.016) and CP + P100 (*p* < 0.001) groups compared with CP. Although CP + P100 showed higher values than CP + P50, this difference was not significant (*p* = 0.653). Relative to Control, CP + P50 exhibited a modest but significant reduction (*p* = 0.044), whereas CP + P100 did not differ significantly (*p* = 0.181), indicating more effective preservation of the primordial follicle pool at the higher propolis dose (Figure 3, Figure 4 and Figure 5A; Table 3).

##### Follicular Subtype Analysis (Primary, Secondary, Tertiary, and Atretic Follicles)

Normality analysis revealed that follicular subtype data did not consistently meet parametric assumptions across all groups; therefore, pairwise comparisons were performed using the Mann–Whitney U test. Compared with the Control group, cisplatin administration (CP) resulted in a significant reduction in primary, secondary, and tertiary follicle counts (primary: *p* = 0.009; secondary: *p* = 0.007; tertiary: *p* = 0.007), accompanied by a significant increase in atretic follicles (*p* = 0.009), indicating marked disruption of folliculogenesis.

Propolis administered alone did not induce significant alterations in follicular subtypes compared with the Control group at either dose (P50 or P100), with no statistically significant differences observed for primary, secondary, tertiary, or atretic follicles (all *p* > 0.05). In contrast, co-administration of propolis with cisplatin significantly ameliorated cisplatin-induced follicular damage. Both CP + P50 and CP + P100 groups exhibited significantly higher primary, secondary, and tertiary follicle counts compared with the CP group (CP vs. CP + P50: primary *p* = 0.018, secondary *p* = 0.027, tertiary *p* = 0.023; CP vs. CP + P100: primary *p* = 0.010, secondary *p* = 0.019, tertiary *p* = 0.007), along with a significant reduction in atretic follicle numbers (CP vs. CP + P50: *p* = 0.047; CP vs. CP + P100: *p* = 0.013).

Direct comparison between CP + P50 and CP + P100 groups revealed no statistically significant differences in primary, secondary, tertiary, or atretic follicle counts (all *p* > 0.05), indicating that both doses exerted comparable protective effects against cisplatin-induced follicular injury (Figure 3, Figure 4 and Figure 5B–E; Table 3).

### 3.2. Biochemical Assessment

#### 3.2.1. Biochemical Evaluation of Oxidative Stress

##### Lipid Peroxidation (MDA)

Lipid peroxidation, reflected by MDA levels, differed significantly among the groups, and one-way ANOVA confirmed a strong overall effect (F(5,27) = 35.65, *p* < 0.001).

Cisplatin (85.52 ± 1.77) significantly increased MDA levels compared with the Control (59.38 ± 3.40; *p* < 0.001), indicating elevated lipid peroxidation. Propolis alone significantly reduced lipid peroxidation, as both the P50 (41.97 ± 4.19) and P100 (36.30 ± 2.62) groups exhibited markedly lower MDA levels than the Control group (*p* = 0.004 and *p* < 0.001, respectively).

**Figure 6:**
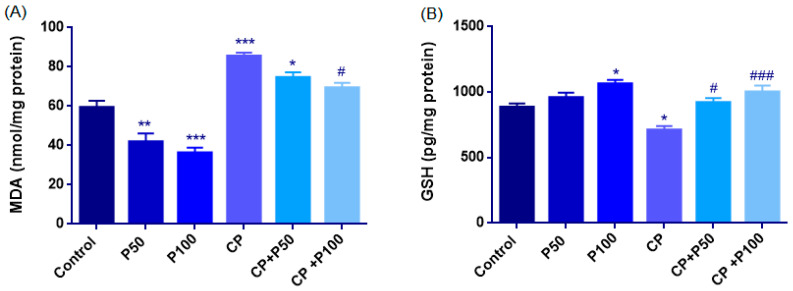
Oxidative stress parameters in the experimental groups. (**A**) Malondialdehyde (MDA) levels (nmol/mg protein) were markedly elevated in the cisplatin (CP) group, whereas propolis (P50, P100) significantly decreased lipid peroxidation both alone and in combination with CP (CP + P50, CP + P100). (**B**) Reduced glutathione (GSH) levels (pg/mg protein) were strongly depleted by CP, while propolis treatment restored GSH concentrations toward or above control values. Data are presented as mean ± SEM. Statistical significance: * *p* < 0.05, ** *p* < 0.01, *** *p* < 0.001 vs. Control; # *p* < 0.05, ### *p* < 0.001 vs. CP.

Propolis co-administration also attenuated cisplatin-induced oxidative damage. MDA levels in the CP + P100 (69.35 ± 2.50) groups were significantly reduced compared with the CP group (*p* < 0.001), but no significant effect CP + P50 (74.66 ± 2.61, *p* = 0.264) group. When CP + P50 was compared with CP + P100, no significant difference was detected (*p* = 0.833). The CP + P50 group showed a significant reduction in MDA levels compared with the control group (*p* = 0.020), whereas the decrease observed in the CP + P100 group was not statistically significant (*p* = 0.205) (Figure 6A, Table 4).

##### Glutathione (GSH)

GSH levels showed a normal distribution (Shapiro–Wilk *p* > 0.05), and homogeneity of variances was confirmed by the Levene test (F(5,27) = 0.363, *p* = 0.869). Accordingly, one-way ANOVA revealed a significant overall group effect (F(5,27) = 9.327, *p* < 0.001).

Cisplatin administration markedly depleted GSH levels; the CP group (710 ± 31 pg/mg) exhibited significantly lower concentrations than the Control group (885 ± 27 pg/mg; *p* = 0.042), indicating cisplatin induces oxidative depletion.

Propolis administration alone enhanced antioxidant capacity. While the P50 group did not differ from the Control (957 ± 38; *p* = 0.703), the P100 group showed a significant elevation (1065 ± 30, *p* = 0.015), suggesting a dose-dependent enhancement of GSH synthesis with higher propolis concentrations.

Co-administration of propolis with cisplatin also substantially restored GSH levels. Both CP + P50 (920± 34, *p* = 0.013) and CP + P100 (1002 ± 48, *p* < 0.001) displayed significantly higher GSH values than the CP group. Moreover, no significant difference was found between the CP + P50 and CP + P100 groups (*p* = 0.636) and the Control group (Figure 6B, Table 4).

#### 3.2.2. Assessment of Inflammatory (TNF-α) and Apoptotic (Caspase-3) Responses

##### TNF-α

TNF-α levels were normally distributed across the groups (Shapiro–Wilk *p* > 0.05); however, the homogeneity of variances assumption was violated (Levene’s test: F(5,27) = 4.813, *p* = 0.003). Therefore, Welch ANOVA was applied, revealing a highly significant overall difference among the groups (Welch’s F(5,12.4) = 268.976, *p* < 0.001).

Cisplatin sharply increased TNF-α levels, rising from 800 ± 53 pg/mg in the Control group to 1942 ± 75 pg/mg in the CP group (*p* < 0.001), indicating a strong inflammatory response. Propolis alone reduced TNF-α levels: P50 (325 ± 21 pg/mg) and P100 (210 ± 13 pg/mg) showed markedly lower levels than those in the control and CP groups (*p* < 0.001).

**Figure 7:**
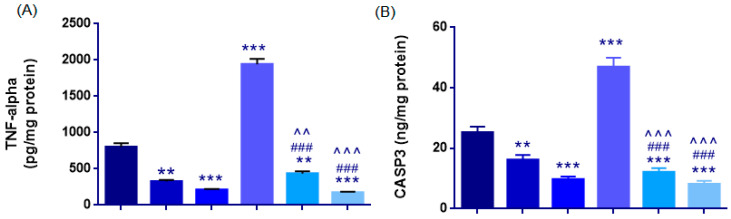
Inflammatory (TNF-α) and apoptotic (Caspase-3) markers in rat ovarian tissue. (**A**) Cisplatin (CP) markedly increased TNF-α levels, whereas propolis (P50, P100) significantly reduced this inflammatory response both alone and in combination with CP (CP + P50, CP + P100). (**B**) Caspase-3 (CASP3) expression was strongly elevated by CP, while propolis treatment suppressed apoptosis in a dose-dependent manner. Data are presented as mean ± SEM. Statistical significance: ** *p* < 0.01, *** *p* < 0.001 vs. Control; ### *p* < 0.001 vs. CP. ^^ *p* < 0.01; ^^^ *p* < 0.001 vs. CP + P50 and CP + P100 groups.

Both CP + P50 (435 ± 31 pg/mg) and CP + P100 (171 ± 15 pg/mg) groups showed profound reductions compared with the CP group (Games-Howell *p* < 0.001). TNF-α levels in both co-treatment groups remained higher than Control (CP + P50: *p* = 0.004; CP + P100: *p* < 0.001), with the 100 mg/kg dose yielding the most significant reduction and outperforming CP + P50 (*p* < 0.001) (Figure 7A, Table 4).

##### Caspase-3 (CASP3)

One-way ANOVA showed a significant group effect on CASP3 levels (F(5,27) = 63.477, *p* < 0.001). The control group exhibited a CASP3 level of 25.20 ± 1.9 ng/mg, while cisplatin markedly elevated caspase-3 expression to 46.94 ± 3.05 ng/mg (*p* < 0.001). Propolis alone significantly reduced CASP3 levels, with both the P50 (16.11 ± 1.7 ng/mg) and P100 (9.67 ± 1.0 ng/mg) groups exhibiting lower values than the Control group (*p* = 0.005 and *p* < 0.001, respectively).

Both CP + P50 (12.14 ± 1.25 ng/mg) and CP + P100 (8.12 ± 1.11 ng/mg) exhibited significantly reduced CASP3 levels compared with CP (*p* < 0.001). No significant difference was observed between the CP + P50 and CP + P100 groups (*p* = 0.544). However, both co-treatment groups retained significantly higher CASP3 levels compared with the Control group (*p* < 0.001 for CP + P50 and *p* < 0.001 for CP + P100), demonstrating that complete normalization to baseline was not achieved (Figure 7B, Table 4).

#### 3.2.3. Biochemical Evaluation of ER Stress Markers

##### CHOP

CHOP levels were normally distributed across groups (Shapiro–Wilk *p* > 0.05), and homogeneity of variances was confirmed by the Levene test (F(5,27) = 1.411, *p* = 0.252). ANOVA showed a significant group effect (F(5,27) = 40.510, *p* < 0.001).

Cisplatin markedly increased CHOP expression, with CP values (618 ± 32 ng/mg) significantly higher than Control (358 ± 14 ng/mg; *p* < 0.001). The P50 group (366 ± 9 ng/mg) was comparable to Control (*p* = 0.999), whereas P100 (260 ± 13 ng/mg) showed a significant decrease (*p* = 0.003).

**Figure 8:**
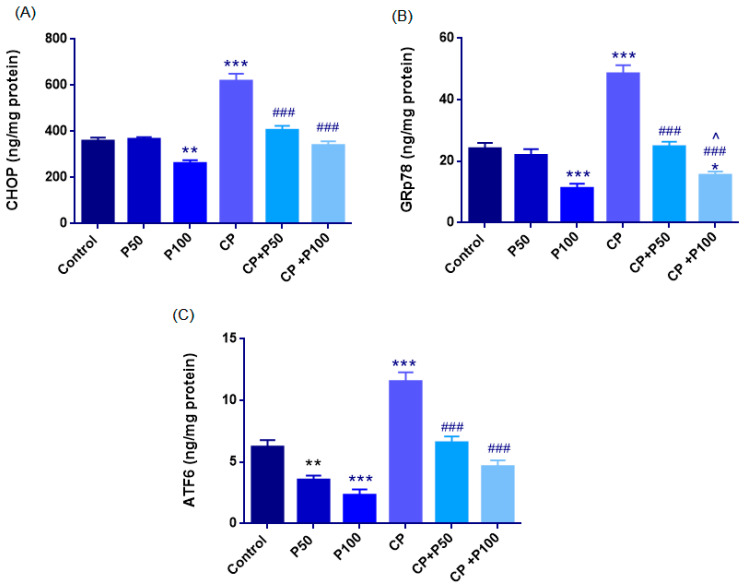
ER stress markers in rat ovarian tissue. (**A**) CHOP levels increased markedly after cisplatin (CP), while propolis (P50, P100) reduced CHOP both alone and in co-treatment (CP + P50, CP + P100). (**B**) GRP78 expression was strongly elevated by CP, whereas propolis decreased GRP78 in a dose-dependent manner and alleviated CP-induced ER stress. (**C**) ATF6 levels increase after CP administration, while propolis—alone or with CP—significantly suppressed ATF6 activation. Statistical significance: * *p* < 0.05, ** *p* < 0.01, *** *p* < 0.001 vs. Control; ### *p* < 0.001 vs. CP; ^ *p* < 0.05 vs. CP + P50 and CP + P100.

Propolis co-treatment reduced cisplatin-induced CHOP elevation, as both CP + P50 (405 ± 19 ng/mg) and CP + P100 (339 ± 17 ng/mg) were significantly lower than CP (*p* < 0.001). No difference was observed between the two co-treatment groups (*p* = 0.114), and their values did not differ from Control, indicating near-baseline restoration (Figure 8A, Table 4).

##### GRP78

GRP78 levels were normally distributed across groups (Shapiro–Wilk *p* > 0.05), and homogeneity of variances was confirmed by the Levene test (F(5,27) = 1.482, *p* = 0.229). A one-way ANOVA revealed a significant overall effect (F(5,27) = 43.080, *p* < 0.001).

The control group exhibited a GRP78 level of 24.12 ± 1.85 ng/mg, whereas cisplatin markedly elevated GRP78 expression to 48.47 ± 2.73 ng/mg, indicating activation of ER-stress-related chaperone response (*p* < 0.001 vs. control). Propolis alone reduced GRP78 levels in a dose-dependent manner: P50 (21.93 ± 2.03 ng/mg) showed values similar to control (*p* = 0.942), while P100 (11.20 ± 1.49 ng/mg) exhibited a significant reduction compared with control groups (*p* < 0.001).

Co-administration of propolis with cisplatin effectively attenuated GRP78 upregulation. Both CP + P50 (24.76 ± 1.56 ng/mg) and CP + P100 (15.50 ± 1.18 ng/mg) groups showed significantly lower GRP78 levels than the CP group (both *p* < 0.001). CP + P100 significantly lowered GRP78 levels compared with the CP + P50 group (*p* = 0.013), indicating a dose-dependent enhancement of the ER-stress-reducing effect. Compared with the Control group, the CP + P50 group showed no significant difference (*p* = 1.000), whereas CP + P100 exhibited significantly higher values (*p* = 0.016) (Figure 8B, Table 4).

##### ATF6

ATF6 levels were normally distributed across groups (Shapiro–Wilk *p* > 0.05), and homogeneity of variances was confirmed by the Levene test (F(5,27) = 0.175, *p* = 0.970). A one-way ANOVA demonstrated a significant overall group effect (F(5,27) = 33.728, *p* < 0.001).

The control group showed an ATF6 concentration of 6.22 ± 0.55 ng/mg, while cisplatin treatment markedly increased ATF6 expression to 11.53 ± 0.74 ng/mg, indicating activation of ER-stress-related signaling (*p* < 0.001 vs. control). Propolis alone reduced ATF6 expression: P50 (3.55 ± 0.35 ng/mg) and P100 (2.32 ± 0.45 ng/mg) both showed significantly lower levels than the control (*p* = 0.007; *p* < 0.001, respectively)

Co-administration of propolis with cisplatin also attenuated ATF6 activation. CP + P50 (6.56 ± 0.51 ng/mg) and CP + P100 (4.64 ± 0.49 ng/mg) groups exhibited significantly reduced ATF6 levels compared with the CP group (both, *p* < 0.001). Moreover, no significant difference was observed between the CP + P50 and CP + P100 groups (*p* > 0.05), and both co-treatment groups showed values comparable to the Control group (*p* > 0.05), indicating that increasing the propolis dose did not produce additional suppression of ATF6 (Figure 8C, Table 4).

## 4. Discussion

This study provides integrated evidence that propolis confers tissue-level protection against cisplatin-induced ovarian injury. Our data indicate that propolis attenuates cisplatin-induced oxidative stress, as reflected by reduced lipid peroxidation and improved antioxidant capacity. These biochemical changes were aligned with reduced vascular congestion, hemorrhage, edema, and inflammatory infiltration, together with significant preservation of primordial, primary, secondary, and tertiary follicle counts and a concomitant reduction in atretic follicles. Additionally, the downregulation of GRP78, ATF6, and CHOP, together with lower caspase-3 activity, was associated with partial attenuation of histopathological features, including follicular degeneration, consistent with modulation of ER-stress-related pathways.

Chemotherapy-induced ovarian toxicity remains a significant concern for reproductive-aged women, with young patients particularly vulnerable to long-term infertility [41,42]. Cisplatin, although central to gynecologic cancer treatment, causes marked ovarian structural and biochemical deterioration [43]. Notably, improvements in vascular congestion, hemorrhage, edema, and inflammatory infiltration were interpreted as indicators of general ovarian tissue protection, whereas follicle-specific outcomes were evaluated based on quantitative follicular counts. In the present study, cisplatin produced severe vascular congestion, hemorrhage, interstitial edema, follicular cell degeneration, and increased inflammatory infiltration. These results align with previous studies demonstrating that cisplatin mainly disrupts ovarian tissue integrity via oxidative stress and apoptosis [12,13,44].

Furthermore, our findings showed that cisplatin treatment significantly reduced the number of primordial, primary, secondary, and tertiary follicles, while increasing atretic follicle counts. These results are consistent with previous studies reporting similar follicular depletion patterns following cisplatin exposure [9,12,45,46].

Propolis treatment effectively mitigated these cisplatin-induced histopathological changes. Both doses, especially 100 mg/kg, reduced hemorrhage, congestion, edema, and improved follicular morphology. These findings are consistent with reports that propolis ameliorates hemorrhage, edema, and follicular degeneration in ischemia–reperfusion injury [47], restores folliculogenesis in Polycystic Ovary Syndrome (PCOS) models [48], and reduces atretic follicles in pesticide-induced ovarian toxicity [18]. Collectively, these findings suggest that propolis may exert cytoprotective effects on ovarian tissue, potentially attenuating chemotherapy-induced gonadotoxicity.

Oxidative stress is widely recognized as a central mechanism in cisplatin-induced ovarian injury, characterized by excessive ROS generation, elevated MDA, and depletion of endogenous antioxidants such as GSH [12,13,43,49]. In this context, the strong antioxidant profile of propolis observed in the present study—reflected by its high phenolic and flavonoid content and supported by increased FRAP activity and low DPPH SC_50_—is consistent with reports showing that samples with higher TPC/TFC exhibit greater antioxidant capacity [50,51,52,53]. Concordantly, cisplatin markedly increased MDA and reduced GSH levels, whereas propolis administration ameliorated these alterations, suggesting attenuation of oxidative stress. Similar antioxidant effects of propolis and its phenolic constituents have been demonstrated in cisplatin-related cardiac, renal, and hepatic injury models [13,54,55,56], as well as in various ovarian damage paradigms [18,45,47,57,58]. Collectively, these findings support that the ovarian protective effect of propolis is closely linked to its phenolic-rich antioxidant capacity.

The oxidative imbalance induced by cisplatin is also a key upstream driver of inflammation. ROS activates TNF-α, leading to vascular dysfunction, and tissue injury [13,59]. Consistent with previous models, cisplatin increased TNF-α markedly [44,60]. Propolis significantly reduced TNF-α levels in our model, reflecting suppressed NF-κB activation. Similar anti-inflammatory effects have been shown in ovarian ischemia–reperfusion injury and LPS-induced inflammation models, where propolis reduced TNF-α immunoreactivity [47,61]. CAPE, a major component of propolis, is a well-known inhibitor of the NF-κB/TNF-α axis [62], supporting our mechanistic interpretation. The attenuation of inflammatory cell infiltration in CP + P100 further confirms effective suppression of ovarian inflammation.

Another major mechanistic contributor to cisplatin toxicity is ER stress. Elevated ROS disrupt ER Ca^2+^ homeostasis and provoke misfolded protein accumulation [63], leading to a self-amplifying ROS–ER stress loop [64,65,66]. GRP78, the master ER chaperone, dissociates from unfolded protein response (UPR) sensors—protein kinase RNA-like endoplasmic reticulum kinase (PERK), inositol-requiring enzyme 1 (IRE1), ATF6—during ER stress, thereby initiating downstream signaling [67]. Cisplatin robustly activates endoplasmic reticulum (ER) stress via the ATF6-CHOP signaling axis, leading to downregulation of the anti-apoptotic protein Bcl-2 (B-cell lymphoma-2), activation of caspases, and a concomitant increase in oxidative injury [66,68]. In vivo, cisplatin elevates CHOP and caspase-12 levels [69], while GRP78/CHOP overexpression is linked to follicular loss and granulosa cell apoptosis [10,70]. In the present study, GRP78, ATF6, CHOP, and caspase-3 were all significantly elevated in the cisplatin group, confirming robust ER stress and ER-mediated apoptosis.

In this study, high-dose propolis was associated with reduced GRP78 and CHOP expression and significant suppression of ATF6 compared with the cisplatin group. Both propolis doses alleviated ER-stress-related markers toward physiological ranges. These findings are consistent with previous evidence indicating that caffeic acid phenethyl ester (CAPE), a major bioactive component of propolis, reduces oxidative stress, restores GSH levels, downregulates GRP78 and CHOP expression [71], and activates Sirtuin/Nrf2 signaling pathways involved in the stabilization of ER Ca^2+^ homeostasis [72].

From a clinical perspective, chemotherapy-induced ovarian toxicity remains a major concern for reproductive-aged women. The present findings suggest that propolis provides tissue-level ovarian protection by attenuating oxidative stress, inflammation, and ER-stress-related apoptotic signaling, key mechanisms involved in chemotherapy-associated gonadotoxicity. Although clinical extrapolation should be approached cautiously, propolis may represent a supportive adjunct strategy for preserving ovarian tissue integrity during chemotherapy. Further preclinical and clinical studies are required to establish its safety, optimal dosing, and translational relevance.

## 5. Conclusions

In the current experimental model, reductions in GRP78, ATF6, CHOP, and caspase-3 activity were observed in association with propolis treatment, suggesting a potential involvement of ER-stress-related pathways and caspase-3–dependent apoptotic signaling in the tissue-level protective effects detected. However, despite substantial biochemical recovery, improvements in follicular structural parameters were comparatively modest, indicating that molecular restoration may precede full architectural recovery of ovarian follicles following cisplatin exposure. Moreover, as the current study is based on biochemical and histopathological associations and does not include functional or pathway-specific validation, definitive mechanistic conclusions regarding direct modulation of the GRP78/ATF6/CHOP axis cannot be drawn. Further functional and pathway-targeted studies are required to clarify the precise role of ER stress signaling in propolis-mediated ovarian protection.

A limitation of the present study is the absence of hormonal assessments related to ovarian endocrine function, such as estradiol, progesterone, or anti-Müllerian hormone; therefore, the reported findings primarily reflect tissue-level outcomes rather than functional endocrine responses.

## Figures and Tables

**Table 1:** Chemical characterization and antioxidant properties of the commercial propolis extract used in the study.

Parameter	Propolis Extract
Total phenolic content (mg GAE/mL)	29.12 ± 0.51
Total flavonoid content (mg QE/mL)	8.29 ± 0.01
FRAP (mg Trolox equivalents/mL)	27.78 ± 0.49
DPPH radical scavenging activity (SC_50_, mg/mL)	0.079 ± 0.001

Sample: The samples used in the study were obtained as water-soluble propolis extracts (O’na Propolis, Okta R&D Engineering Services Industry and Trade Ltd. Co., Trabzon, Turkey). The propolis concentration of the extract was 40%, and following procurement, the samples were stored under refrigerated conditions. Abbreviations: total phenolic content; TFC, total flavonoid content; FRAP, ferric reducing antioxidant power; DPPH, 2,2-diphenyl-1-picrylhydrazyl.

**Table 2:** Histopathological damage scores of all study groups.

	Vascular Congestion	Hemorrhage	Follicular Cell Degeneration	Edema	Inflammatory Cell Infiltration
Control	0 [0–0.25]	0 [0–0.25]	0 [0–1]	0 [0–0.25]	0 [0–0.25]
P50	0 [0–0.25]	0 [0–0.25]	0.50 [0–1]	0 [0–0.25]	0 [0–0.25]
P100	0 [0–1]	0 [0–0.25]	0.50 [0–1]	0 [0–0.25]	0 [0–0.25]
CP	3 [2.25–3] **	2 [2–2.75] **	1 [1–1.75] *	2 [1.25–2.75] **	1 [1–1.75] *
CP + P50	1 [1] **, ##	1 [1] **, ##	1 [1–1.5] *	1 [0–1] #	0 [0–1]
CP + P100	0 [0–1] ##,^	0 [0–0.25] ##,^^	1 [1–1.25] *	0.50 [0–1] #	0 [0–1] #

Statistical significance: * *p* < 0.05, ** *p* < 0.01 vs. Control group; # *p* < 0.05, ## *p* < 0.01 vs. CP group; ^ *p* < 0.05, ^^ *p* < 0.01 vs. CP + P50 and CP + P100 groups. Values are presented as median [interquartile range].

**Table 3:** Quantitative assessment of follicle counts across experimental groups.

	Primordial Follicle	Primary Follicles	Secondary Follicle	Tertiary Follicle	Atretic Follicle
Control	24 ± 0.6	9.33 ± 0.66	4.00 ± 0.26	3.67 ± 0.21	1.50 ± 0.22
P50	22.83 ± 0.7	8.50 ± 0.67	3.83 ± 0.31	3.33 ± 0.21	1.67 ± 0.21
P100	23 ± 0.9	8.83 ± 0.54	4.03 ± 0.36	3.33 ± 0.42	1.50 ± 0.22
CP	11.25 ± 0.8 **	3.00 ± 0.41 **	1.75 ± 0.25 **	1.75 ± 0.25 **	9.75 ± 0.85 **
CP + P50	17.20 ± 1.3 **,#	6.60 ± 0.74 *, #	3.00 ± 0.32 *,#	2.80 ± 0.25 *,#	7.20 ± 0.58 **,#
CP + P100	19.50 ± 1.4 *, #	7.83 ± 0.48, #	3.33 ± 0.33 #	3.33 ± 0.21 ##	6.50 ± 0.43 **,#

Statistical significance: * *p* < 0.05, ** *p* < 0.01 vs. Control group; # *p* < 0.05, ## *p* < 0.01 vs. CP group; Values are presented as mean ± SEM.

**Table 4:** Oxidative Stress, ER-Stress, Inflammatory, and Apoptotic Markers in Rat Ovarian Tissue Across Experimental Groups.

Parameters	Control	P50	P100	CP	CP + P50	CP + P100
MDA (nmol/mg)	59.38 ± 3.39	41.97 ± 4.19 **	36.30 ± 2.61 ***	85.52 ± 1.76 ***	74.66 ± 2.61 *	69.35 ± 2.50 #
GSH (pg/mg)	885 ± 27	957 ± 38	1065 ± 30 *	710± 31 *	920± 34 ##	1002 ± 48 ###
CHOP (ng/mg)	358 ± 14	366 ± 9	260 ± 13 **	618 ± 32 ***	405 ± 19 ###	339 ± 17 ###
GRp78 (ng/mg)	24.12 ± 1.85	21.93 ± 2.03	11.20 ± 1.49 ***	48.47 ± 2.73 ***	24.76 ± 1.56 ###	15.50 ± 1.18 ###,^
ATF6 (ng/mg)	6.22 ± 0.55	3.55 ± 0.35 **	2.32 ± 0.45 ***	11.53 ± 0.74 ***	6.56 ± 0.51 ###	4.64 ± 0.49 ###
TNF-α (pg/mg)	800 ± 53	325 ± 21 **	210 ± 13 ***	1942 ± 75 ***	435 ± 31 **,###, ^^	171 ± 15 ***,###,^^^
CASP3 (ng/mg)	25.20 ± 1.9	16.11 ± 1.7 **	9.67± 1 ***	46.94 ± 3.05 ***	12.14 ± 1.25 ***,###,^^^	8.12 ± 1.11 ***,###,^^^

Statistical significance: * *p* < 0.05, ** *p* < 0.01, *** *p* < 0.001 vs. Control group; # *p* < 0.05, ## *p* < 0.01, ### *p* < 0.001 vs. CP group; ^ *p* < 0.05, ^^ *p* < 0.01, ^^^ *p* < 0.001 vs. CP + P50 and CP + P100 groups. Data are expressed as mean ± standard error of the mean (SEM).

## Data Availability

The data that support the findings of this study are not publicly available due to ethical reasons, but they are available from the corresponding author upon request.
